# From the Editor’s Desk

**Published:** 2014-04

**Authors:** Paul A Brink

**Affiliations:** Department of Internal Medicine, Faculty of Health Sciences, University of Stellenbosch and Tygerberg Hospital, Tygerberg

As I expertly skirt some potholes in a town centre through which we are travelling, I ponder the upcoming edition of the journal. An editorial needs to be written and time is running short. I cannot afford to lose a tyre or worse, a wheel or axle today. In my head I peruse the articles while being mindful of the road.

An interesting take on the Baker hypothesis hails from Poland (Zamecznik *et al.*, page 73). I wonder what roads in Poland are like. The group assessed six- to 10-year-old children and stratified them into groups of appropriate for gestational age (AGA) and small for gestational age (SGA). They further sub-stratified into symmetric (everything small, but proportionately so) and asymmetric (low birth weight, but appropriate length and head circumference). I imagine a malnourished, thin baby with a visibly large head and eyes.

This is a rather interesting study. At school-going age there was no difference in anthropometric measurements, yet the average blood pressure differed between the groups, being higher in the SGA group, with some intragroup differences caused by the type of growth stunting. They refer to articles supporting their findings (Woelk *et al.*^1^ and Thame *et al.*^2^). I must make time to have a look at these.

I skirt another pothole. What are roads like, I wonder, in Lagos, Nigeria, the source of our next article? The economy I believe has been exploding, and gross domestic product, a measure of wealth creation, has surpassed that of South Africa. Nigeria has been a source of sophisticated work on cardiac function, which has recently been published in the journal. In this issue, Ale and co-workers studied left ventricular mass and QT dispersion (QT_d_) in prehypertensive individuals. The QT interval is of special interest to me, and QT_d_ has its traps, called *slaggate* in my home language, literally translated as potholes. This is encapsulated in one of the articles referred to (Sahu *et al.*^3^). Ale and co-workers concluded that echocardiography, albeit expensive, may be a good tool for risk stratification in prehypertension, but that the usefulness of the ECG is limited.

A thud, as I encounter a pothole, breaks my train of thought. Yes, I am still in South Africa. This makes me think of the review by Naidoo *et al.* (page 83) on diabetes mellitus and the use of sulphonylureas; the good and bad aspects. Looking at the authorship, it is not quite free of industrial interest, yet it is timely. The authors used the opportunity to introduce some new kids on the block, albeit expensive and not time-tested, the di-peptidyl peptidase IV inhibitors, glucagon-like peptide analogues and sodium glucose co-transporter inhibitors. Use of the last is limited in a resource-constrained environment, but they suggest that, where hypoglycaemia is not a problem, there may be a place for their use where public safety is concerned, such as bus drivers with diabetes mellitus.

And so the journey continues. I hope I can recover the train of thought when home. Yes, once again there are a few articles from Turkey, one from Ankara and one from Çanakkale (I must go and look on my map where these cities are). These studies documented arrhythmias in neonates (Binnetoglu *et al.*, page 58), cardiovascular involvement in Bechet’s disease (Ulusan *et al.*, page 63) and the effect of topical hypothermia on postoperative inflammatory markers (Kadan *et al.*, page 67).

From South Africa, not surprisingly, as this country has more HIV-positive individuals than any other (http://www.who.int/en/), Nel and Naidoo (page 50) present an article on endocarditis, HIV and echocardiographic findings. A technical article (Steyn *et al.*, page 44) draws attention to the fact that in radionucleotide imaging, different software does not measure ejection fractions equally. Once again, the print issue is complemented by online-only publication of a case report.

Ah, I am home at last.
